# Amino Acid and Carotenoid Profiles of *Chlorella vulgaris* During Two-Stage Cultivation at Different Salinities

**DOI:** 10.3390/bioengineering12030284

**Published:** 2025-03-13

**Authors:** Ana S. Pinto, Carolina Maia, Sara A. Sousa, Tânia Tavares, José C. M. Pires

**Affiliations:** 1 LEPABE—Laboratory for Process Engineering, Environment, Biotechnology and Energy, Faculty of Engineering, University of Porto, Rua Dr. Roberto Frias, 4200-465 Porto, Portugal; up202008182@edu.fe.up.pt (A.S.P.); up201704692@edu.fe.up.pt (C.M.); up201806617@edu.fe.up.pt (S.A.S.); tsgtavares@fe.up.pt (T.T.); 2 ALiCE—Associate Laboratory in Chemical Engineering, Faculty of Engineering, University of Porto, Rua Dr. Roberto Frias, 4200-465 Porto, Portugal

**Keywords:** amino acids, biomass productivity, biotechnological applications, β-carotene, lutein, neoxanthin, two-stage cultivation, violaxanthin, zeaxanthin

## Abstract

Microalgae are valuable sources of bioactive compounds. However, their production requires strategies to enhance metabolic responses. This study explores how *Chlorella vulgaris* responds to different salinity conditions using a two-stage cultivation strategy, assessing the change in amino acid and carotenoid content on microalgae over time. First, microalgae were cultivated under optimal conditions, followed by exposure to different salinity levels (150 mM and 300 mM NaCl). Growth kinetics, nutrient uptake, and biochemical composition were analysed, revealing distinct salinity-induced responses. Similar specific growth rates were achieved across all assays, while nitrate removal improved under salinity and phosphate uptake decreased. Amino acid profiling showed significant declines in the content of several compounds and carotenoid content also presented declining trends, although moderate salinity mitigated degradation in key pigments. Principal component analysis identified high correlations between amino acids and carotenoids contents, forming groups of compounds with similar variations. These findings contribute to a better understanding of the salinity-induced response of *C. vulgaris*, offering insights for biotechnology applications. By optimising cultivation conditions, salinity could enhance bioactive compound retention, supporting the development of sustainable microalgae-based products.

## 1. Introduction

Microalgae are photosynthetic microorganisms capable of synthesising high-energy organic compounds from inorganic materials, using light as an energy source; they are responsible for releasing around 50% of the world’s O_2_ annually [1]. Microalgae are abundant in terrestrial and aquatic ecosystems, are extremely diverse, have fast growth rates, and possess a remarkably versatile metabolism, resulting in a broad range of applications [2,3]. Considering environmental applications, the global energy crisis and depletion of fossil-fuel reserves have triggered significant research interest in alternative energies. Microalgae produce lipids that can be converted into biodiesel, saccharides that can be used to produce bioethanol or biogas, and starch that can be biodegraded into bio-hydrogen. Simultaneously, they are cultivated in bioremediation strategies for wastewater treatment (via N and P consumption) and mitigation of CO_2_ emissions [1,4,5].

Complementing all this, microalgae are commercially used for human nutrition, as well as animal and aquatic feed, serving as an inexpensive protein source [6,7]. Furthermore, they are a major source of bioactive compounds, including essential amino acids and carotenoids, which have substantial relevance for food applications. Amino acids derived from microalgae, particularly essential ones that cannot be synthesised by the human body (e.g., leucine, lysine, and valine), are critical for maintaining cellular functions, muscle growth, and overall health [8]. Their presence in microalgal biomass makes these microorganisms an excellent candidate for fortifying food and feed formulations, particularly in protein-deficient regions [9]. Also, carotenoids such as lutein, β-carotene, and zeaxanthin possess potent antioxidant properties. These compounds play vital roles in human health, including improving vision, reducing inflammation, and preventing oxidative stress-related diseases [10]. Their natural origin and bioactivity position microalgae as an attractive alternative to synthetic additives in the functional food and nutraceutical industries [3,11]. Notably, the carotenoid market is rapidly growing [12], with microalgae being increasingly viewed as a sustainable and efficient source for their production.

A two-step cultivation strategy can be applied to promote the production of compounds of interest. First, microalgae are cultivated in optimal conditions to maximise biomass productivity, and then specific changes in culture conditions are applied to induce the production of target compounds [13,14]. They can be broadly classified as nutritional (e.g., depletion of essential nutrients) or environmental ones (e.g., changes in pH, salinity, light intensity, temperature, or reactor configurations) [15,16,17,18]. Salinity, in particular, causes various biochemical and bioenergetic changes [2,19]. It disrupts cell ion homeostasis, triggering a temporary increase in cytosolic Ca^2+^ concentration, which in turn regulates effector membrane proteins to restore balance. Additionally, other messenger molecules activate response signals, including reactive oxygen species (ROS), which are abiotic stress indicators. However, excessive ROS accumulation leads to lipid peroxidation, membrane deterioration, DNA and protein damage, and the inhibition of photosynthesis and growth [2,20]. Microalgae may accumulate antioxidant compounds, such as carotenoids, to counteract elevated ROS levels, which can scavenge free radicals as a defence mechanism [19]. These biomolecules are essential in ideal conditions for light harvesting during photosynthesis and protection against photo-oxidative damage [21].

Among the over 40,000 known microalgae species, *Chlorella vulgaris* is one of the most notable ones [22]. This eukaryotic microorganism, the first discovered with a well-defined nucleus, grows in freshwater and has maintained genetic stability for over 2.5 billion years. Its high protein content (over 55% dry weight) introduced it as an unconventional food source. Additionally, *C. vulgaris* produces all essential amino acids required for human nutrition, making it a valuable resource for addressing dietary protein deficiencies. Furthermore, it is a rich source of carotenoids, including lutein and β-carotene, widely recognised for their antioxidant properties and health benefits, such as vision enhancement and protection against oxidative stress [23,24]. Beyond its nutritional value, *C. vulgaris* demonstrates exceptional adaptability and metabolic versatility, thriving in various environmental conditions and exhibiting a fast growth rate. These traits make it a preferred species for large-scale cultivation in biotechnological applications. Given these characteristics, *C. vulgaris* was chosen for this study.

The novelty of this study lies in its specific focus. As far as it is known, this is the first two-stage cultivation in batch mode of *C. vulgaris*, an abundant and economically viable microalga species, focusing on salinity-induced responses regarding amino acid and carotenoid content over time during the stress stage. Along with similar studies, this work provides the foundational knowledge to determine optimal conditions for large-scale microalgae production units. It is worth emphasising that studying salinity changes in a freshwater microalga is increasingly relevant due to global freshwater shortages [2]. Overcoming the major challenge of large-scale freshwater microalgae production or capitalising on salinity-induced responses is crucial for advancing seawater cultivation practises. Besides this, the particular attention given to salinity conditions in recent research and why it was chosen as the focus of this work can be related to the industrial scalability of its application. While, for instance, inducing temperature changes in larger bioreactors can be demanding regarding energy and controllability, salt addition is easy and inexpensive. Naturally, sodium chloride, as the most abundant salt in seawater, is the common choice for studies on salinity-induced responses, which have been conducted in the past to improve lipid, pigment, and protein production [2,25].

In this study, *C. vulgaris* was cultivated using a two-stage approach, inducing different salinity conditions in the second stage and without a pre-harvesting step between stages. Growth kinetics and biomass productivity were evaluated alongside nutrient consumption. Lastly, amino acid and carotenoid profiles were investigated throughout the different cultivation stages as well as under salinity conditions. The assumption is that salinity induces adaptive metabolism mechanisms to retain bioactive compounds. In detail, moderate salinity levels are expected to stabilise carotenoid content and induce specific amino acid profiles. A coherent comprehension of these responses is crucial for developing optimised microalgal culture strategies for biotechnological processes.

## 2. Materials and Methods

### 2.1. Microalgae

The microalga *C. vulgaris* CCAP 211/11B used in this study was provided by Culture Collection of Algae and Protozoa (CCAP, Scotland, UK). The microalga was inoculated under axenic conditions in Erlenmeyer flasks and maintained at room temperature under a continuous light supply of 6.5 μmol m^−2^ s^−1^. Stirring was carried out using a Unimax 1010 orbital shaker (Heidolph, Schwabach, Germany) set to 120 rotations per min (rpm). Culture was kept in the modified OECD (Organization for Economic Cooperation and Development) with the following composition (per litre): 250 mg NaNO_3_, 12 mg MgCl_2_⋅6H_2_O, 18 mg CaCl_2_⋅2H_2_O, 15 mg MgSO_4_⋅7H_2_O, 45 mg KH_2_PO_4_, 0.08 mg FeCl_3_⋅6H_2_O, 0.1 mg Na_2_EDTA⋅2H_2_O, 0.185 mg H_3_BO_3_, 0.415 mg MnCl_2_⋅4H_2_O, 3 × 10^−3^ mg ZnCl_2_, 1.5 × 10^−3^ mg CoCl_2_⋅6H_2_O, 0.01 × 10^−3^ mg CuCl_2_⋅2H_2_O, 7 × 10^−3^ mg Na_2_MoO_4_⋅2H_2_O, and 500 mg NaHCO_3_.

### 2.2. Culture Conditions and Experimental Design

The microalga *C. vulgaris* was inoculated in Schott round clear borosilicate glass bottles with 1 L capacity each. The initial biomass concentration was 0.33 ± 0.02 g_dw_ L^−1^. All bioreactors were maintained at a constant temperature of 20 °C in a Panasonic MLR-352 PE climate chamber under a continuous light supply of 132 ± 14 µmol m^−2^ s^−1^, provided by a horizontal LED lamp. The growth medium used was the modified OECD medium, with all concentrations doubled. Aeration, which promotes medium homogenisation and provides CO_2_, was achieved through continuous bubbling of atmospheric air (with a CO_2_ content of 0.04%) at the bottom of the flasks using air pumps (Sicce Airlight 3300, Pozzoleone, Italy). The air was filtered through 0.22 µm nylon membrane filters (Specanalitica, Cascais, Portugal) at a 1.7 L min^−1^ flow rate.

Two independent experiments were conducted to test the two intended conditions, each lasting 15 consecutive days. For each one, seven bottles of positive control (inoculated culture medium with no salt added to promote optimal microalgae growth) and seven salinity-sample bottles were analysed.

The cultures were grown in batch mode, with the bottles positioned at equal distances from the light source. Luminosity was measured on day 0 of the experiment using a portable photo/radiometer (Delta OHM HD2102.2; Delta ohm srl, Padua, Italy). Flasks were randomly reassigned to available positions daily to minimise uneven light intensity distribution. The positioning and respective measurements are represented in Figure S1.

Water evaporation from the medium was balanced daily by adding distilled water to restore the initial volume. Sampling for nutrient analysis was conducted on days 0, 1, 2, 4, 8, 11, and 15, with samples collected alternately from three bioreactors to form biological triplicates. On days 8, 11, and 15, duplicate control bioreactors and duplicate salinity sample bioreactors were harvested for amino acid and carotenoid extraction. A seventh bioreactor was inoculated for each condition to account for potential errors or accidents.

### 2.3. Salinity Treatment

On day 8 of each experiment, sodium chloride (NaCl) was added to all seven salinity sample bottles. Two concentrations were tested, namely 150 mM NaCl (8.77 g per 1 L flask) for the first assay (A150) and 300 mM NaCl (17.53 g per 1 L flask) for the second assay (A300). No salt was added to the control flasks (C-A150 and C-A300).

### 2.4. Analytical Methods: pH and Microalgal Growth Monitoring

Daily pH measurements were performed using a Consort C6010 electrochemical analyser (Brussels, Belgium). Optical density (OD) measurements were conducted at 680 nm using a Shimadzu UV mini-1240 spectrophotometer (Shimadzu Europe, Duisburg, Germany), with duplicate readings for all flasks. Previously determined calibration curves were used to convert OD readings into biomass concentrations.

Microalgal growth rates (*μ*, d^−1^) were calculated during the exponential phase using Equation (1):

(1) $$\ln\frac{X_{2}}{X_{1}}=\mu \left(t_{2}-t_{1}\right)$$

where *X*_2_ and *X*_1_ represent biomass concentrations (g_dw_ L^−1^) in the final (*t*_2_) and initial instant (*t*_1_) of the exponential growth phase, respectively. Average biomass productivity (*P_X,avg_*, g_dw_ L^−1^ d^−1^) was calculated using Equation (2):

(2) $$P_{X,avg}=\frac{X_{f}-X_{0}}{t_{f}-t_{0}}$$

where *X*_0_ and *X_f_* are the initial and final biomass concentrations, and *t*_0_ and *t_f_* are the start and end times of the experiment.

### 2.5. Analytical Methods: Nutrient Concentration Monitoring

For nutrient concentration monitoring, 5 mL samples were collected to monitor nitrogen and phosphorus removal from the medium on days 0, 1, 2, 4, 8, 11, and 15. These experimental points are considered to be representative of the typical nutrient removal curve [26]. These samples were centrifuged in an Eppendorf 5810 R centrifuge (Hamburg, Germany) at 4000 rpm for 10 min and 20 °C. The supernatant was then filtered with 0.22 µm cellulose acetate membrane syringe filters (Whatman plc, Maidstone, UK) and frozen at −20 °C for storage until a simultaneous analysis of all samples.

Phosphate–phosphorus (PO_4_-P) analysis followed a procedure described in the literature [27] based on the reaction of inorganic phosphate with ammonium molybdate, producing a phomolybdate complex that absorbs at 820 nm. The absorbance was read in a Synergy HT 96-well microplate reader (Biotek Instruments, Inc., Winooski, VT, USA). On the other hand, nitrate–nitrogen (NO_3_-N) concentration was determined following the methodology described by Collos et al. [28]. The absorbance was read in a UV-6300PC Double Beam spectrophotometer (VWR, Amadora, Portugal).

The nutrient concentrations were obtained in both analyses through previously determined calibration curves. From those values, the quantification of nitrogen and phosphorus removal by microalgae was based on three parameters, namely removal efficiency (*RE*, %) (Equation (3)), mass removal (*MR*, mg L^−1^) (Equation (4)), and average removal rate (*RR*, mg L^−1^ d^−1^) (Equation (5)). In these equations, *S*_0_ and *S_f_* (in mg L^−1^) are the nutrient concentrations at the beginning and end of each experiment, respectively, and (*t_f_* − *t*_0_) represents its duration in d.

(3) $$RE=\frac{S_{0}-S_{f}}{S_{0}}\times 100$$

(4) $$MR=S_{0}-S_{f}$$

(5) $$RR=\frac{S_{0}-S_{f}}{t_{f}-t_{0}}$$

### 2.6. Analytical Methods: Harvesting and Extraction

Cell harvesting was performed on days 8, 11, and 15 of each experiment. The entire volume of the bioreactors was centrifuged using a Beckman Avanti™ J-25 (Beckman Coulter, Brea, CA, USA) centrifuge at 8000 rpm for 10 min at 20 °C. The resulting pellet was transferred to Petri dishes, sealed with perforated parafilm, and lyophilised using a 6K Benchtop freeze dryer (VirTis, New York, NY, USA) under vacuum conditions. After lyophilisation, the moisture-free samples were transferred to Falcon tubes, labelled, and stored under refrigeration until further analysis.

The carotenoid extraction method followed recommendations from Pagels et al. [29], which identified single solvent extraction as a reliable technique for pigment recovery. Acetone was chosen as the solvent for its eco-friendliness and low toxicity. Despite its relatively lower efficiency and longer processing time than alternative methods, acetone’s compatibility with downstream analysis makes it an optimal choice for this study [30,31].

Since *C. vulgaris* does not excrete carotenoids into the growth medium, extraction requires efficient cell disruption. A total of 100 mg of dry biomass (prepared in triplicate) was combined with 0.1 mm glass microspheres in a 6:1 (*w*/*w*) ratio for each sample. A known concentration of trans-β-Apo-8′-apo-carotenal (170 mg L^−1^; ExtraSynthase) was added as an internal standard to account for analyte losses during the extraction process. Subsequently, 4 mL of acetone was added to each sample. Cell disruption was achieved using a Precellys^®^ Evolution Touch homogeniser (Bertin Technologies, Montigny-le-Bretonneux, France) programmed for six 30 s cycles at 8000 rpm, with 45 s pauses between cycles. Samples were kept on ice between cycles to prevent degradation of the thermosensitive carotenoids [32,33]. An additional 4 mL of acetone was added, and the homogenisation cycle was repeated twice.

The extracts were centrifuged using a GZ-1580R centrifuge (Gyrozen, Daejeon, Republic of Korea) at 5000× *g* for 10 min at 5 °C. The supernatant was filtered through 0.45 µm PTFE filters (VWR) and dried under a gentle nitrogen stream using an SBHCONC/1 Sample Concentrator (Stuart, Stone, UK). The dried extracts were saturated with nitrogen to avoid oxidation, stored in a desiccator under low humidity, and kept in the dark until further analysis. Before injection into analytical equipment, the residue was resuspended in a mixture of acetone and ethyl acetate (9:1, *v*/*v*).

### 2.7. Analytical Methods: Amino Acid and Carotenoid Quantification

Following the method outlined by Cohen and Deantonis [34], the amino acid profile was ascertained. In total, 20 mg of the freeze-dried samples were weighed in a 10 mL glass hydrolysis tube, and 3 mL of hydrochloric acid solution (HCl, 6 M) containing 0.5% (*w*/*v*) phenol was added. To seal the tubes under vacuum, nitrogen gas was pumped into the vials. The hydrolysis process was carried out using the reference method AOAC 982.30 at 110 °C for 24 h [35]. Following hydrolysis, 0.2 mL from the hydrolysate was neutralised with NaOH 6 M, and the final volume was adjusted with borate buffer (0.1 M) to 1 mL. Centrifugation was used to separate the undissolved particles for 1 min at 13,000 rpm. An AccQ-Tag derivatization kit (Waters^TM^, Milford, MA, USA) was used to derivatize amino acids. In short, 5 μL of the sample was combined with 5 μL of internal standard 200 μM (D-norleucine). To this mixture was added 70 μL borate buffer and 20 μL derivatisation agent. After the amino acids were derivatised, they were subjected to high-performance liquid chromatography (HPLC) analysis using a Waters Alliance 2695 (Milford, MA, USA) fitted with an AccQ-Tag Amino Acids C18 Column (4 µm, 150 × 3.9 mm) (Waters, Wexford, Ireland), a photodiode array (Waters, Milford, MA, USA) set to spectrum scanning within the range of *λ* = 210–600 nm, a fluorescence detector (Waters, Milford, MA, USA) set to *λ_Ex_* = 250 nm and *λ_Em_* = 395 nm (PMT = 100), and a column heater (Waters, USA) set to 37.0 °C. Following injection (5 µL), chromatographic separation was carried out using a combination of three eluents, comprising a patented aqueous buffer (eluent A, Waters, Milford, MA, USA), milli-Q water for mobile phase B, and acetonitrile for mobile phase C. A 1 mL min^−1^ flow rate was used for elution, and the following chromatographic separation was achieved: 0 min (A, 100%); 0.5 min (A, 99 percent; B, 1 percent); 18 min (A, 95%; B, 5%); 19 min (A, 91%; B, 9%); 28 min (A, 83%; B, 17%); 35 min (B, 60%; C, 40%); and 38 min (A, 100%) for a total analysis duration of 47 min. The Amino Acid Food and Feed Standard Kit (Waters, USA) was used for the calibration. It contains 21 amino acids, such as alanine (ALA), α-aminobutyric acid (AABA), arginine (ARG), aspartic acid (ASP), cystein (CYS), cysteic acid (CYA), glutamic acid (GLU), L-glycine (GLY), L-histidine (HIS), L-isoleucine (ILE), L-leucine (LEU), lysine (LYS), methionine (MET), methionine sulfone (MetO), phenylalanine (PHE), L-proline (PRO), L-serine (SER), taurine (TAU), L-threonine (THR), L-tyrosine (TYR), and valine (VAL). Seventeen of those amino acids were quantified (CYS was not detected in all performed assays) using a five-point calibration curve (ranging from 5 to 250 μM of each analyte) using D-norleucine as the internal standard (IS).

To identify and quantify carotenoids, the extracts were also analysed using HPLC, the same performed for amino acid determination, where a 4 × 250 mm Purospher Star RP-18e (5 μm) column (Merck) was used as the stationary phase, coupled with a 5 μm 4.0 × 20 mm pre-column. The mobile phase consisted of HPLC-grade ethyl acetate as solvent A and a mixture of acetonitrile and water (9:1) as solvent B. The column was kept at a temperature of 25 ± 2 °C and a pressure of 3000 bar (column heater, Waters, USA), and the flow rate was fixed at 1 mL min^−1^. During the 55 min elution period, the solvent gradient looked as follows: between 0 and 31 min (0–60% A); 31–36 min (60% A); 36–38 min (60–100% A); 38–43 min (100% A); 43–50 min (100% A); and 50–55 min (0% A). Spectral data were collected over the range of 250 to 750 nm for all peaks. Compound identification was achieved by comparing the retention times and spectra with authenticated standards. HPLC-grade standards for lutein, zeaxanthin, and β-carotene were used.

Calibration curves for each analyte, based on seven calibration points, were prepared. The lutein calibration curve was used for the quantification of violaxanthin and neoxanthin. An additional calibration curve was constructed for the internal standard. A minimum of three independent experiments was performed for each analysis. Using the calibration curve parameters, the limit of quantification (LOQ) and limit of detection (LOD) were calculated. The results are expressed in milligrammes per gram of dry algae (mg g_dw_^−1^).

### 2.8. Statistical Analysis

For each parameter, the mean value and standard deviation were determined. Statistical treatment was performed using an ANOVA followed by the Tukey–Kramer multiple comparison method. The results were considered statistically different if *p* < 0.05.

Principal component analysis (PCA) is a multivariate statistical technique used to reduce the dimensionality of large datasets while retaining as much variability as possible [36,37,38]. It transforms the original variables into a new set of uncorrelated variables called principal components (PCs), ordered by the amount of variance they explain. PCA was applied to group amino acids and carotenoids based on their temporal variation in concentrations. The number of principal components to retain was determined using the Kaiser criterion. It selects PCs with eigenvalues greater than 1, indicating that they account for a significant portion of the data variance.

## 3. Results

### 3.1. Biomass Growth and Productivity

**Figure 1:**
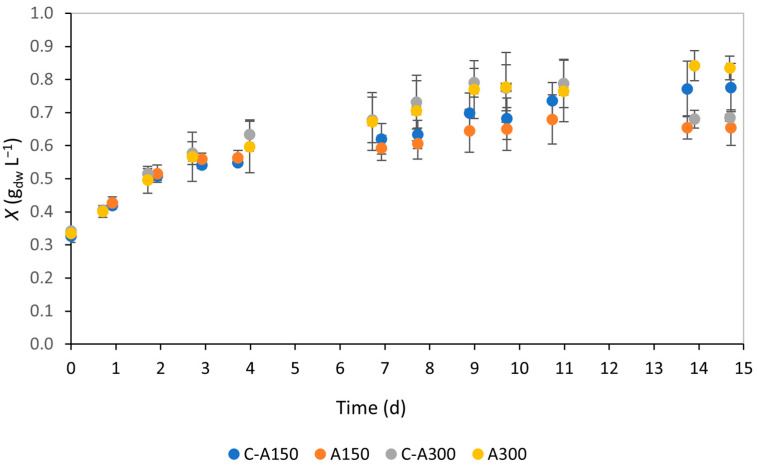
*Chlorella vulgaris* biomass concentrations of assays A150 (150 mM NaCl) and A300 (300 mM NaCl) and respective controls (to which no salt was added, C-A150 being the control for the assay at 150 mM NaCl and C-A300 the control for the assay at 300 mM NaCl) throughout an observed period of 15 d.

The evolution of biomass concentration throughout the 15 d assays, A150 and A300, is shown in Figure 1. Visual inspection of the growth curves reveals that all four follow similar trends during the first week of the experiment when the microalgae are in their exponential growth phase. In subsequent stages, slight divergences emerge in the A300 assays, where biomass concentration exceeds the control cultures from day 14 onward. By the end of the experiment, all cultures appeared to have reached the stationary phase, with biomass values stabilising as expected in batch cultures; however, no cultures entered the death phase during the observation window.

While visual observations provide an overview of growth patterns, they do not offer precise, comparative conclusions. Therefore, two parameters were used to quantify growth, namely the specific growth rate (*µ*, d^−1^) (Equation (1)) and average biomass productivity (*P_X,avg_*, mg_dw_ L^−1^ d^−1^) (Equation (2)). The results for these parameters are summarised in Table 1. The data reveal no statistically significant difference in specific growth rate in the presence of salt (*p* > 0.05). This lack of difference is likely because only the first three experimental points were considered for *µ* calculation, focusing on the exponential growth phase when the growth curve exhibits its steepest slope. Since both the assays and respective controls overlapped significantly in this phase, similar growth rates were expected, and the results demonstrate good reproducibility. Regarding average biomass productivity, calculations consider the entire experimental period by comparing the initial and final data points, so these variations should reflect the salinity conditions tested. A slight decrease in the *P_X,avg_* was observed in the A150 assay, while an increase was noted in the A300 assay compared to their respective controls (Table 1). Although the *P_X,avg_* between the A150 assay and the respective control was statistically significant, the observed difference was minimal (*p* = 0.047).

Regarding pH, which is known to fluctuate with microalgal growth, values were monitored throughout the experiment (Figure S2). For both assays and controls, pH increased during the exponential growth phase before stabilising around 10 or slightly decreasing in the later stages. The observed increase during the exponential growth phase correlates with CO_2_ consumption during photosynthesis. Its stabilisation around 10 aligns with findings suggesting that *C. vulgaris* achieves optimal biomass productivity in alkaline conditions (pH 10–10.5) [39,40].

### 3.2. Nutrient Consumption

**Figure 2:**
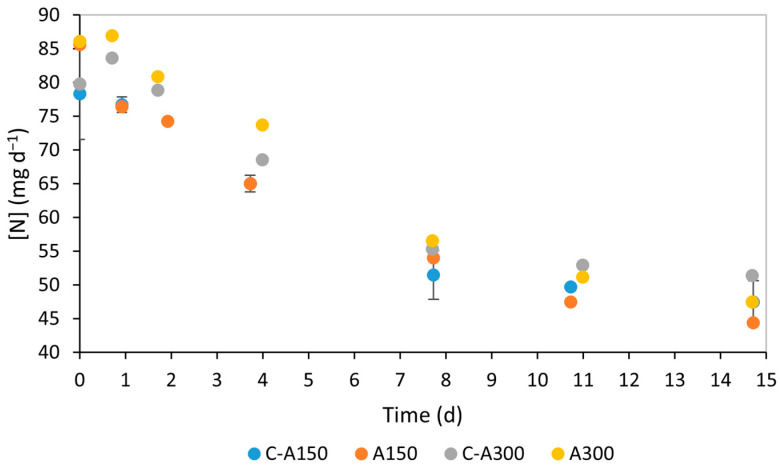
Nitrate concentrations in *Chlorella vulgaris* culture medium of assays A150 (150 mM NaCl) and A300 (300 mM NaCl) and respective controls (with no salt added, C-A150 being the control for the assay at 150 mM NaCl and C-A300 the control for the assay at 300 mM NaCl) throughout an observed period of 15 d.

**Figure 3:**
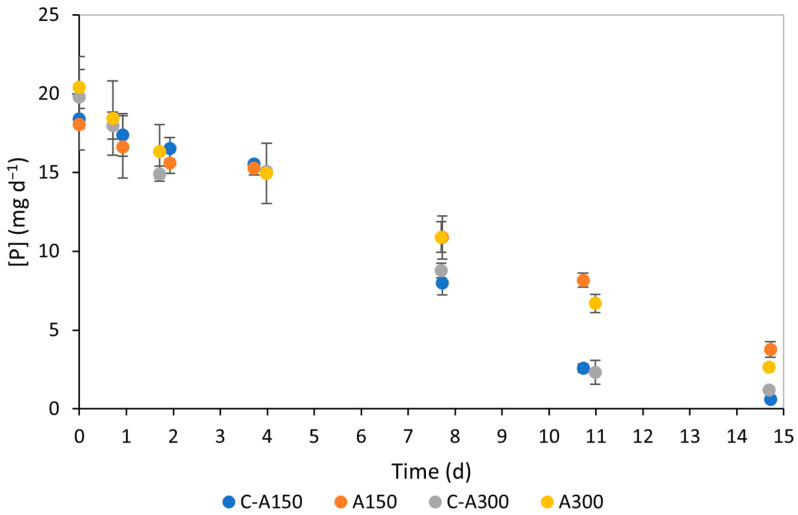
Phosphate concentrations in *Chlorella vulgaris* culture medium of assays A150 (150 mM NaCl) and A300 (300 mM NaCl) and respective controls (with no salt added, C-A150 being the control for the assay at 150 mM NaCl and C-A300 the control for the assay at 300 mM NaCl) throughout an observed period of 15 d.

Figure 2 and Figure 3 show the evolution of NO_3_-N and PO_4_-P concentrations in the medium over the 15 d of the performed assays. The progressive decrease in NO_3_-N and PO_4_-P concentration, visible in Figure 2 and Figure 3, respectively, indicates the microalgae’s effective consumption of these nutrients. Concerning NO_3_-N, no significant differences were observed in the temporal variation in its concentration in the performed assays. For PO_4_-P, similar concentrations were observed during the first stage of the experiments. However, some differences became apparent in the second phase of the experiments when the samples were exposed to different salinity conditions. Specifically, on day 11, A150 and A300 assays exhibited higher phosphate concentrations than their respective controls, indicating less efficient nutrient consumption.

Regarding nitrogen quantification, nitrate (NO_3_^−^) was used as the oxidised form of nitrogen, as it is the sole nitrogen source in the modified OECD medium. Table 2 summarises the parameters used to quantify nitrogen consumption by *C. vulgaris* in the performed assays. Initial nitrogen concentrations showed no significant differences (*p* > 0.05). Considering the *S*_0_ values for nutrients and their molecular masses, the N/P molar ratio was estimated to be nine, which falls within the range known to support effective nitrate removal [41]. An increase in the mass of nitrogen removed was observed in both assays compared to the control, indicating higher nitrogen removal efficiency. At 150 mM NaCl and 300 mM NaCl, the RE for nitrogen increased by 10% compared to the respective control. This suggests a positive effect of salt on nitrate removal by *C. vulgaris*. The MR and RR parameters, which depend on the same variables, showed similar trends, with A150 values still relatively close to those of the control.

Table 3 summarises five key parameters that characterise nutrient removal, allowing for an assessment of the impact of salinity-induced response on phosphorus uptake. As expected, no statistically significant differences (*p* > 0.05) were observed in the initial PO_4_-P concentrations, which aligned with the theoretically predicted value based on the culture medium composition (90 mg L^−1^ of KH_2_PO_4_, equating to 20.5 mg L^−1^ of PO_4_-P). This conversion assumes a 1:1 ratio between KH_2_PO_4_ and P. *S_f_* values were higher for both assays than for their respective controls, indicating that more phosphorus remained unconsumed in the culture medium after 15 d. Notably, the effect was more pronounced in the A150 assay compared to the A300 assay. As anticipated from the differences in *S*_0_ and *S_f_*, RE decreased in the presence of salt, with reductions of 17.7% at 150 mM NaCl and 7.0% at 300 mM NaCl. Further analysis of the other parameters (MR and RR) revealed this reduction only in the A150 assay.

### 3.3. Amino Acid Profile

Table 4 shows the amino acid profile in *C. vulgaris* cultures over time in the two experimental setups (A150 and A300) and their respective controls (C-A150 and C-A300) (an example of a chromatogram is shown in Figure S3). Sixteen amino acids were analysed. The results revealed distinct temporal trends, offering insights into how salinity affects amino acid metabolism and its implications for microalgal cultivation. In the control cultures, most amino acids showed a steady or slight increase over the 15-day experimental period. This pattern reflects the natural biosynthesis and accumulation of amino acids as part of normal cellular metabolism during exponential and stationary growth phases. For instance, ASP in C-A150 has a positive temporal trend. Similarly, GLU in the same control also increased steadily. In contrast, the salinity-induced cultures exhibited different trends. In A150, ASP content presented a negative temporal trend and in A300, ASP showed an even sharper decline over the same period. A similar pattern was also observed for GLU.

### 3.4. Carotenoid Profile

Table 5 presents the changes in carotenoid content over time in microalgae subjected to different salinity conditions (an example of a chromatogram is shown in Figure S4). The carotenoids analysed include neoxanthin, violaxanthin, lutein, zeaxanthin, and β-carotene. Lutein was the most abundant carotenoid in all samples, consistent with its role as a major antioxidant in *C. vulgaris* [42]. Concerning temporal trends, neoxanthin exhibited a steady decline over time in the control samples. Under salinity conditions, the A150 assay showed a slightly steeper decline, suggesting that 150 mM NaCl had no protective effect on neoxanthin stability. However, in the A300 assay, the decline in neoxanthin content was lower than that of the corresponding control, indicating that 300 mM NaCl mitigated its natural degradation during the stationary phase. This suggests that moderate salinity at 300 mM NaCl can slow the degradation of neoxanthin potentially by activating protective biochemical pathways. The other carotenoids’ content showed similar behaviour.

The results highlight contrasting effects of salinity on carotenoid stability and accumulation. While 150 mM NaCl generally accelerated the decline of most carotenoids, 300 mM NaCl demonstrated a beneficial effect by reducing the natural degradation rates of neoxanthin, violaxanthin, lutein, and β-carotene. These findings suggest that salinity at 300 mM NaCl, particularly during the stationary growth phase, supports carotenoid retention.

### 3.5. Principal Component Analysis

PCA was applied to the amino acid and carotenoid content in *C. vulgaris* under different salinity conditions [36,37,38]. Table 6 shows the rotated factor loadings (RFLs) that represent the main contributions of the original variables (amino acids and carotenoid contents) on the principal components (PCs). The first four PCs accounted for a high percentage of the total variance (>90%), with PC1 and PC2 being the most significant. The grouping of variables along these components provides insights into their correlations and the biochemical processes underlying their production and degradation [36].

## 4. Discussion

### 4.1. Biomass Growth and Productivity

This study evaluated the impact of salinity on *C. vulgaris* growth, nutrient consumption, and carotenoid and amino acid profiles. To evaluate microalgal growth, the growth rate and productivity were analysed. Regarding specific growth rate, Church et al. [43] reported slightly higher values (0.27–0.29 d^−1^) in batch cultures over 8 d, while Pandit et al. [44] obtained lower rates (0.105 ± 0.089 d^−1^) after 15 d of cultivation. Luangipat and Chisti [45] observed rates of 0.359 d^−1^ in 2 L bottles and 0.649 d^−1^ in 1 L bottles, with the difference attributed to the area-to-volume ratio affecting light capture. Despite variations in experimental conditions and *C. vulgaris* strains, the present results (0.22–0.24 d^−1^) fall within the range of values present in the literature. Regarding average biomass productivity, Pandit et al. [44] found higher values (31 ± 3 mg L^−1^ d^−1^) at 100 mM NaCl compared to the control (28 ± 4 mg L^−1^ d^−1^), but a decrease (24 ± 4 mg L^−1^ d^−1^) was observed at 300 mM NaCl. The control *P_X,avg_* values in the present study are comparable to those in Pandit et al.’s work [44], but the response to salt differs. The results obtained by El-fayoumy et al. [46] are also of special pertinence for comparison to this work since a two-stage cultivation strategy was applied, despite the larger time intervals—a 25-day vegetative stage was followed by a 15-day salinity stage. Similarly to this study, biomass productivity increased from the control cultures (38.25 ± 0.66 mg L^−1^ d^−1^) to the cultures subjected to 15 g L^−1^ of NaCl (44.83 ± 1.37 mg L^−1^ d^−1^). Although both *P_X,avg_* values are higher than those in Table 1, a similar increasing tendency was observed for an also similar NaCl concentration of 300 mM (or 17.53 g L^−1^). An increase followed by a decrease in productivity with rising salt concentrations is a common observation in other studies [45,47,48,49]. As *C. vulgaris* is a freshwater microalga, this suggests a critical point of salinity beyond which growth slows due to energy reallocation towards osmotic acclimation rather than reproduction. High Na^+^ concentrations can also be toxic to cells, causing them to expend energy to pump excess ions into the medium, reducing toxicity [49]. The present results suggest that the highest salinity tested here (A300) was below this toxicity threshold, as productivity increased rather than being inhibited. This increase could also be attributed to the positive effect of salinity in controlling biological contaminants. Shen et al. [50] and Ishika et al. [51] reported that increasing salinity in the medium reduces the contamination issue, which may explain the increased productivity at 300 mM NaCl.

### 4.2. Nutrient Consumption

Microalgae require nutrients to grow, with nitrogen and phosphorus assimilation closely tied to biomass production. Nitrogen RE was higher when salt was added to the culture medium. As for phosphorus, the reduction in RE for the A150 assay correlates with the decline in average biomass productivity. However, for the A300 assay, although biomass productivity increased, phosphorus removal followed the opposite trend. A more likely explanation is that salinity reduced the microalgae’s capacity to uptake phosphorus, a trend also observed in higher plants [52] even though the biomass continued to grow. Comparable studies support these findings. Biliani and Manariotis [47], who tested NaCl concentrations ranging from 0 to 35 g L^−1^, observed a clear increase in nitrate consumption with increasing salinity, while phosphorus removal decreased. Their best-performing samples reached near-zero concentrations of both nutrients by the end of the experiment. In contrast, the control sample maintained a nitrate concentration near 40 mg_N_ L^−1^, corresponding to approximately 60% removal efficiency. Similarly, Luangipat and Chisti [45] found an increase in nitrate consumption from 6.83 mg g_dw_^−1^ L^−1^ in freshwater to 10.28 mg g_dw_^−1^ L^−1^ in full seawater (40 g L^−1^ NaCl). Furthermore, the study showed a slight decrease in phosphate consumption as NaCl concentration increased from their freshwater control value (0.189 mg g_dw_^−1^ L^−1^) to the intermediate medium (0.155 mg g_dw_^−1^ L^−1^). Church et al. [43] hypothesised that while the Na⁺ ion facilitates phosphorus uptake in green algae, this effect is limited to lower salinities (e.g., 0.1 g L^−1^ NaCl). Conversely, for nitrate removal, Daneshvar et al. [53] suggested that the bioadsorption of this compound could play a role, with salinity enhancing bioadsorption on both live and dead cells, which may explain the observed increase in nitrate uptake in this study.

### 4.3. Amino Acid Profile

The amino acid profile reveals how salinity influences their production and accumulation, offering insights into its impact on microalgal cultivation. In A150, ASP content decreased over time, with an even sharper decline in A300, following a similar pattern to GLU. These declines in amino acid content could be associated with the disruption of normal biosynthesis or the increased utilisation of amino acids. For instance, ASP and GLU are precursors for other metabolic compounds (indirectly involved in chlorophyll production), and their reduction under salinity conditions may indicate their diversion into pathways supporting osmotic adjustment or other adaptive mechanisms [54]. On the other hand, PRO accumulation under salinity conditions has been widely reported [55], since PRO plays a crucial role in mitigating osmotic and oxidative pressure caused by the uncontrolled generation of varied reactive oxygen species promoted by high salinity levels, thereby maintaining cellular osmotic balance. This effect could be related to the PRO biosynthesis pathway in *C. vulgaris*, a metabolic adaptation that is a cycle also involving glutamate [55]. However, the current study does not corroborate these findings directly, as PRO content in microalgae decreased over time.

The amino acid contents observed in this study fall within the range reported in the literature, although variations exist due to differences in experimental conditions. Ursu et al. [56] reported the amino acid profile of *C. vulgaris*, and the presented values were close to those achieved in this study. The trends observed here align with those findings, particularly the salinity-induced decline in ASP and GLU. This trend was also observed by Bhatnagar et al. [57] when studying the effect of salinity for 14 d on the composition of the microalga *Pseudochlorella pringsheimii*. The authors observed a decrease in the content of all the amino acids tested when a salinity of 5 g/L was introduced. For example, GLU, also the main amino acid, decreased from a content of (10.40 ± 0.04) mg/100 mg of biomass to (5.91 ± 0.03) mg/100 mg of biomass. Thus, after 14 d under salinity conditions, the total amino acid content was equal to (10.12 ± 0.15) mg/100 mg of biomass for the test with 5 g/L NaCl in contrast to the result of (23.69 ± 0.38) mg/100 mg of biomass for the control (no NaCl added).

The observed decline in most amino acids under salinity conditions suggests some metabolic impact in *C. vulgaris* [53,57,58]. Salinity imposes osmotic and ionic pressure, disrupting cellular homeostasis and triggering adaptive responses. Amino acids like PRO are upregulated to counteract osmotic pressure, while others like ASP and GLU may be diverted into salinity-induced pathways or utilised for energy production. The reduction in amino acid biosynthesis could also result from impaired nitrogen assimilation, a known consequence of salinity environments [54,59].

The differences between A150 and A300 highlight the dose-dependent effects of salinity. While both salinity levels negatively impacted amino acid content overall, the effects were more pronounced at 300 mM NaCl. For example, SER in A150 decreased by 0.19 mg g_dw_^−1^ d^−1^, whereas in A300, the decline was steeper at 0.27 mg g_dw_^−1^ d^−1^. Similarly, VAL and THR exhibited more significant reductions in A300.

### 4.4. Carotenoid Profile

The results regarding carotenoid content offer insights into the impact of salinity on carotenoid biosynthesis and degradation, particularly in the stationary growth phase. Hynstova et al. [33] evaluated the carotenoid profile of seven commercial samples of dried *C. vulgaris* by high-performance thin-layer chromatography (HPTLC). Although the methods differ, it is interesting to note that the carotenoids found in this study were reported for all samples. Lutein content varied from 553.5 ± 0.2 μg g^−1^ to 2221.1 ± 0.9 μg g^−1^. The highest zeaxanthin content found was 1283.4 ± 0.5 μg g^−1^, while the lowest was 73.89 ± 0.03 μg g^−1^. β-carotene concentrations ranged from 101.58 ± 0.04 μg g^−1^ to 728.1 ± 0.3 μg g^−1^. All of these ranges comprise the carotenoid content found in the present work, attesting to the reasonability of the results. These authors also mentioned that lutein and zeaxanthin are stereoisomers, which structurally vary only in the position of one double bond. Thus, separating these pigments from each other was difficult, which could have caused some uncertainty in their quantification.

The results show that salinity has contrasting effects on carotenoid stability. While 150 mM NaCl generally accelerated carotenoid degradation, 300 mM NaCl helped preserve neoxanthin, violaxanthin, lutein, and β-carotene, especially during the stationary phase, likely due to osmotic adaptation or enhanced antioxidant activity. These findings align with studies by Ali et al. [19] and El-fayoumy et al. [46], which reported that salinity-induced response can promote carotenoid retention by triggering biochemical pathways associated with oxidative defence. However, the lack of significant carotenoid accumulation under salinity conditions in this study may reflect the relatively low severity of the tested salinity levels (150–300 mM NaCl). Higher salinity levels or the combination of salinity with other factors (e.g., high light intensity) may further enhance carotenoid production.

### 4.5. Salinity Impact on Productivity, Nutrient Consumption, and Compound Production

**Figure 4:**
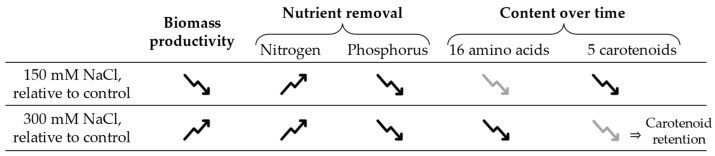
Summary of the main trends observed in the results of the present study. Upward arrows represent increases and downward arrows represent declines relative to the control, and within the same column, the distinction between grey and black arrows refers to less pronounced and more pronounced variations, respectively.

In general, the main temporal trends observed for the various indicators of microalgal metabolism are summarised in a simplified way in Figure 4, where the two presently studied salinity values can be compared to the respective control, according to their impacts on biomass productivity, nutrient removal, and content in amino acids and carotenoids. The figure shows that compared to the control where no NaCl was added to the cultures, biomass productivity is decreased by 150 mM of NaCl but increased in 300 mM NaCl conditions. Nitrogen removal is enhanced by both salinity values, and phosphorus removal is hindered. Amino acids show negative temporal trends with both NaCl concentrations, with 300 mM showing the largest (negative) deviation from the control. Regarding carotenoids, both temporal trends are also negative; however, their absolute value is larger than the control at 150 mM NaCl but lower than the control at 300 mM, suggesting an effective carotenoid retention.

### 4.6. Principal Component Analysis

PC1 is characterised by strong negative loadings for several amino acids, including ASP, GLU, ALA, LYS, and VAL, among others. These amino acids have a central role in the metabolic network and primary metabolism of *C. vulgaris* [54,59,60]. The grouping of amino acids in PC1 with strong negative loadings suggests salinity-induced coordinated alterations [61].

The grouping of GLY, TYR, PHE, and most carotenoids (neoxanthin, violaxanthin, lutein, and β-carotene) in PC2 suggests a metabolic connection between these compounds. As precursors for phenolic and antioxidant compounds [62], TYR and PHE may influence carotenoid biosynthesis under salinity-induced oxidative response. Similarly, GLY may support protein synthesis or act as a building block for enzymatic pathways involved in carotenoid production. The strong negative correlation between ARG and PRO in PC3 reflects a metabolic trade-off in nitrogen utilisation. While ARG supports general nitrogen metabolism [54], PRO is an osmoprotectant under salinity conditions [55]. This result reflects the interconnected roles of ARG and PRO in microalgae metabolism and highlights the adaptive strategies of *C. vulgaris* in managing nitrogen resources [63]. Zeaxanthin’s distinct behaviour and strong association with PC4 underscore its specialised role in photoprotection [64]. Unlike other carotenoids, zeaxanthin’s dynamics are closely linked to the xanthophyll cycle, which dissipates excess light energy as heat [65]. This unique function likely separates it metabolically from pigments like lutein and β-carotene, which serve broader antioxidant roles.

The presented amino acid and carotenoid profiles under different conditions offer opportunities to optimise *C. vulgaris* cultivation. Further targeted studies on metabolome or gene expression could help shed light on the metabolic interplay between amino acids and carotenoids, increasing the robustness of the conclusions and correlations. Combining salinity conditions with other factors, such as light intensity or nutrient limitation, could enhance the production of specific metabolites like PRO and carotenoids. Additionally, targeting metabolic pathways associated with PC1 could improve nitrogen utilisation efficiency, promoting growth and salinity tolerance.

## 5. Conclusions

The findings reveal that salinity-induced response, particularly at 300 mM NaCl, can enhance biomass productivity by mitigating some environmental issues, such as biological contamination. This response highlights the feasibility of employing salinity as a strategic tool for optimising large-scale cultivation.

The analysis of amino acid profiles revealed notable reductions in ASP and GLU. Carotenoid biosynthesis also exhibited intriguing trends. While the accumulation of carotenoids was not substantially enhanced, moderate salinity (300 mM NaCl) slowed the degradation of pigments like neoxanthin and lutein. This suggests that salinity triggers protective pathways that support pigment stability, particularly during the stationary growth phase. Such responses could be harnessed to improve the antioxidant potential of microalgae-derived products.

PCA provided insights into the simultaneous changes observed in amino acids and carotenoids, underlining the potential for tailoring cultivation strategies to optimise the production of bioactive compounds. To further validate the trends observed and whether they can be explained by changes in cell metabolism, it is recommended that complementary metabolism studies are conducted, along with other experiments considering more extreme salinity conditions. Overall, this research lays a strong foundation for the sustainable exploitation of *C. vulgaris*, paving the way for further exploration into the synergistic effects of salinity and other factors to maximise metabolite yields in industrial settings.

## Figures and Tables

**Table 1:** *Chlorella vulgaris* specific growth rates and average biomass productivity values for assays A150 (150 mM NaCl) and A300 (300 mM NaCl) and respective controls (with no salt added, C-A150 being the control for the assay at 150 mM NaCl and C-A300 the control for the assay at 300 mM NaCl) throughout 15-day experiments.

Assay	*µ* (d^−1^)	*P_X,avg_* (mg_dw_ L^−1^ d^−1^)
C-A150	0.23 ± 0.02 ^a^	29 ± 3 ^a^
A150	0.22 ± 0.02 ^a^	23 ± 3 ^b^
C-A300	0.241 ± 0.001 ^a^	23 ± 1 ^b^
A300	0.23 ± 0.01 ^a^	34 ± 3 ^c^

*μ*: specific growth rate; *P_X,avg_*: average microalgal cell productivity. Values are presented as the mean ± standard deviation obtained from seven independent experiments. Within the same column, average values sharing at least one common letter are not statistically different (*p* > 0.05).

**Table 2:** Quantification parameters of NO_3_-N consumption by *Chlorella vulgaris* during an observed period of 15 d for assays A150 (150 mM NaCl) and A350 (300 mM NaCl) and respective controls (with no salt added, C-A150 being the control for the assay at 150 mM NaCl and C-A300 the control for the assay at 300 mM NaCl).

Assay	*S*_0_ (mg L^−1^)	*S_f_* (mg L^−1^)	*RE* (%)	*MR* (mg L^−1^)	*RR* (mg L^−1^ d^−1^)
C-A150	78 ± 7 ^a^	47 ± 3 ^a^	39 ± 2 ^a^	36 ± 4 ^a,c^	2.6 ± 0.3 ^a^
A150	85.7 ± 0.5 ^a^	44 ± 3 ^a^	49.5 ± 0.4 ^b^	42.4 ± 0.6 ^b^	2.88 ± 0.04 ^b^
C-A300	80 ± 6 ^a^	51 ± 9 ^a^	35 ± 1 ^a^	32 ± 2 ^a^	2.2 ± 0.2 ^c^
A300	86 ± 4 ^a^	47.5 ± 0.3 ^a^	45 ± 3 ^c^	39 ± 4 ^b,c^	2.76 ± 0.05 ^a,b^

*MR*: mass removal; *RE*: removal efficiencies; *RR*: average removal rate; *S*_0_: initial substrate concentration; *S_f_*: final substrate concentration. Values are presented as the mean ± standard deviation from three independent experiments. Within the same column, average values sharing at least one common letter are not statistically different (*p* > 0.05).

**Table 3:** Quantification parameters of PO_4_-P consumption by *Chlorella vulgaris* during an observed period of 15 d for assays A150 (150 mM NaCl) and A350 (300 mM NaCl) and respective controls (with no salt added, C-A150 being the control for the assay at 150 mM NaCl and C-A300 the control for the assay at 300 mM NaCl).

Assay	*S*_0_ (mg L^−1^)	*S_f_* (mg L^−1^)	*RE* (%)	*MR* (mg L^−1^)	*RR* (mg L^−1^ d^−1^)
C-A150	18.4 ± 0.6 ^a^	0.60 ± 0.05 ^a^	96.7 ± 0.3 ^a^	17.8 ± 0.6 ^a^	1.21 ± 0.04 ^a^
A150	18 ± 2 ^a^	3.8 ± 0.5 ^b^	79 ± 2 ^b^	15.2 ± 0.5 ^b^	1.03 ± 0.03 ^b^
C-A300	20 ± 2 ^a^	1.2 ± 0.1 ^c^	94 ± 1 ^a^	19 ± 2 ^a^	1.3 ± 0.1 ^a^
A300	20 ± 2 ^a^	2.6 ± 0.2 ^d^	87 ± 2 ^c^	18 ± 2 ^a^	1.2 ± 0.1 ^a^

*MR*: mass removal; *RE*: removal efficiencies; *RR*: average removal rate; *S*_0_*:* initial substrate concentration; *S_f_*: final substrate concentration. Values are presented as the mean ± standard deviation obtained from three independent experiments. Within the same column, average values sharing at least one common letter are not statistically different (*p* > 0.05).

**Table 4:** Content of 16 amino acids (in mg g_dw_^−1^) in *Chlorella vulgaris* cultures in assays A150 (150 mM NaCl) and A300 (300 mM NaCl) and respective controls (with no salt added, C-A150 being the control for the assay at 150 mM NaCl and C-A300 the control for the assay at 300 mM NaCl) at days 8 (T8), 11 (T11), and 15 (T15) of the 15-day experiments.

Amino Acid	Assay	Amino Acid Content (mg g_dw_^−1^)			TT (mg g_dw_^−1^ d^−1^)
		T8	T11	T15	
ASP–Aspartic acid	C-A150	7.23 ± 0.39 ^a^	7.45 ± 0.33 ^a^	8.41 ± 0.48 ^c^	0.17 ± 0.04
	A150	10.56 ± 0.10 ^b^	8.88 ± 0.47 ^c^	6.74 ± 0.37 ^a,d^	−0.55 ± 0.03
	C-A300	6.77 ± 0.85 ^a,d^	6.16 ± 0.49 ^d^	7.40 ± 0.43 ^a^	0.09 ± 0.07
	A300	6.88 ± 0.20 ^a,d^	3.60 ± 0.13 ^e^	3.82 ± 0.19 ^e^	−0.45 ± 0.10
SER–L-Serine	C-A150	4.39 ± 0.18 ^a,e^	4.57 ± 0.15 ^a,b^	4.03 ± 0.22 ^c,e^	−0.06 ± 0.03
	A150	4.93 ± 0.15 ^b^	4.52 ± 0.31 ^a,b,e^	3.64 ± 0.12 ^c^	−0.19 ± 0.02
	C-A300	4.37 ± 0.53 ^a,e^	3.62 0.21 ^c^	4.40 ± 0.12 ^a,e^	0.01 ± 0.06
	A300	4.59 ± 0.06 ^a,b^	2.34 ± 0.13 ^d^	2.54 ± 0.12 ^d^	−0.27 ± 0.07
GLU–Glutamic acid	C-A150	8.49 ± 0.46 ^a,c^	9.48 ± 0.55 ^d,g^	10.04 ± 0.65 ^e,g^	0.21 ± 0.06
	A150	12.42 ± 0.11 ^b^	10.53 ± 0.51 ^e^	7.76 ± 0.28 ^a^	−0.67 ± 0.04
	C-A300	7.94 ± 0.48 ^a^	7.87 ± 0.23 ^a^	9.93 ± 0.49 ^e,g^	0.30 ± 0.07
	A300	9.03 ± 0.25 ^c,d^	4.43 ± 0.12 ^f^	5.02 ± 0.19 ^f^	−0.59 ± 0.15
GLY–L-Glycine	C-A150	5.34 ± 0.38 ^a,c,d^	5.52 ± 0.32 ^a,c^	4.12 ± 0.17 ^b,c,d^	−3.37 ± 0.99
	A150	5.17 ± 0.12 ^a,b,c,d^	4.69 ± 0.15 ^a,b,c,d^	4.30 ± 0.25 ^b,c,d^	−6.71 ± 1.32
	C-A300	6.04 ± 0.16 ^a^	4.85 ± 0.23 ^a,b,c,d^	4.89 ± 0.41 ^a,b,c,d^	−3.34 ± 1.33
	A300	5.45 ± 0.68 ^a,c,d^	3.67 ± 0.29 ^b^	3.85 ± 0.32 ^b,d^	−2.25 ± 0.81
HIS–L-Histidine	C-A150	9.56 ± 0.64 ^a,f^	9.69 ± 0.60 ^a,f^	11.54 ± 0.29 ^d^	2.61 ± 0.53
	A150	14.39 ± 0.89 ^b^	11.04 ± 0.83 ^d^	10.46 ± 0.27 ^a,d^	−1.42 ± 0.36
	C-A300	7.37 ± 0.52 ^c^	6.25 ± 0.49 ^c,g^	8.91 ± 0.47 ^f^	1.37 ± 0.63
	A300	7.09 ± 0.32 ^c^	2.12 ± 0.18 ^e^	5.00 ± 0.15 ^g^	−0.56 ± 0.39
THR–L-Threonine	C-A150	3.79 ± 0.25 ^a,b^	4.00 ± 0.26 ^a,b^	3.73 ± 0.20 ^b^	−0.01 ± 0.03
	A150	4.24 ± 0.09 ^a^	3.75 ± 0.26 ^b^	3.10 ± 0.10 ^c^	−0.16 ± 0.02
	C-A300	3.71 ± 0.40 ^b^	3.02 ± 0.22 ^c^	3.79 ± 0.21 ^a,b^	0.02 ± 0.05
	A300	3.65 ± 0.14 ^b^	2.10 ± 0.12 ^d^	2.32 ± 0.16 ^d^	−0.19 ± 0.05
ARG–Arginine	C-A150	17.84 ± 1.90 ^a^	17.71 ± 1.46 ^a^	19.19 ± 1.44 ^a^	0.23 ± 0.17
	A150	13.87 ± 0.66 ^b^	13.17 ± 1.80 ^b^	13.57 ± 0.77 ^b^	−0.01 ± 0.15
	C-A300	9.26 ± 0.74 ^c^	14.60 ± 0.03 ^b^	25.81 ± 1.83 ^e^	2.38 ± 0.16
	A300	9.84 ± 0.60 ^c^	6.45 ± 0.52 ^d^	8.90 ± 0.68 ^c,d^	−0.07 ± 0.18
ALA–Alanine	C-A150	2.57 ± 0.09 ^a,f^	2.71 ± 0.01 ^a^	2.74 ± 0.11 ^a^	0.02 ± 0.01
	A150	3.42 ± 0.19 ^b^	3.11 ± 0.15 ^c^	2.36 ± 0.09 ^d,f^	−0.15 ± 0.02
	C-A300	2.70 ± 0.20 ^a^	2.23 ± 0.10 ^d^	2.78 ± 0.13 ^a^	0.01 ± 0.03
	A300	2.78 ± 0.11 ^a^	1.43 ± 0.09 ^e^	1.70 ± 0.02 ^g^	−0.16 ± 0.05
PRO–L-Proline	C-A150	3.83 ± 0.23 ^a,e^	3.92 ± 0.25 ^a,e^	3.64 ± 0.21 ^e^	−0.03 ± 0.02
	A150	6.89 ± 0.12 ^b^	6.09 ± 0.40 ^d^	4.27 ± 0.39 ^a,c,e^	−0.39 ± 0.04
	C-A300	4.74 ± 0.46 ^c,f^	4.32 ± 0.13 ^a,c^	4.03 ± 0.29 ^a,e^	−0.10 ± 0.03
	A300	6.18 ± 0.28 ^d^	4.78 ± 0.11 ^c,f^	5.14 ± 0.33 ^f^	−0.14 ± 0.06
TYR–L-Tyrosine	C-A150	5.32 ± 0.39 ^a^	5.29 ± 0.31 ^a^	3.85 ± 0.34 ^d,e^	−0.24 ± 0.05
	A150	4.42 ± 0.69 ^b,c,e^	4.05 ± 0.01 ^c,d,e^	3.78 ± 0.36 ^d,e^	−0.09 ± 0.05
	C-A300	5.12 ± 0.28 ^a,b^	4.73 ± 0.31 ^a,b,c^	4.21 ± 0.09 ^c,d,e^	−0.13 ± 0.03
	A300	5.31 ± 0.44 ^a^	3.61 ± 0.43 ^d^	3.56 ± 0.23 ^d^	−0.24 ± 0.07
VAL–Valine	C-A150	3.84 ± 0.09 ^a,c^	3.99 ± 0.24 ^a,c^	4.01 ± 0.14 ^a,c^	0.02 ± 0.02
	A150	4.96 ± 0.26 ^b^	4.63 ± 0.28 ^b^	3.80 ± 0.14 ^a,c^	−0.17 ± 0.02
	C-A300	4.21 ± 0.49 ^a^	3.65 ± 0.16 ^c^	4.53 ± 0.18 ^b^	0.08 ± 0.05
	A300	4.77 ± 0.10 ^b^	2.46 ± 0.15 ^d^	2.95 ± 0.11 ^d^	−0.25 ± 0.09
MET–Methionine	C-A150	1.64 ± 0.15 ^a,d^	1.80 ± 0.09 ^a,b^	1.26 ± 0.19 ^c,d^	−0.06 ± 0.03
	A150	2.13 ± 0.20 ^b^	1.95 ± 0.18 ^a,b^	1.66 ± 0.15 ^a,d^	−0.07 ± 0.02
	C-A300	1.90 ± 0.33 ^a,b^	1.69 ± 0.08 ^a^	1.68 ± 0.14 ^a^	−0.03 ± 0.03
	A300	2.01 ± 0.22 ^a,b^	0.98 ± 0.09 ^c^	0.97 ± 0.24 ^c^	−0.14 ± 0.03
ILE–L-Isoleucine	C-A150	0.39 ± 0.04 ^a,c^	0.42 ± 0.05 ^c^	0.31 ± 0.03 ^b,f^	−0.01 ± 0.01
	A150	0.35 ± 0.04 ^a,b^	0.31 ± 0.03 ^b,f^	0.28 ± 0.03 ^d,f^	−0.01 ± 0.01
	C-A300	0.30 ± 0.03 ^b,d,f^	0.24 ± 0.01 ^d^	0.26 ± 0.02 ^d,f^	0.00 ± 0.00
	A300	0.29 ± 0.03 ^b,d,f^	0.00 ± 0.00 ^e^	0.17 ± 0.03 ^g^	−0.02 ± 0.01
LYS–Lysine	C-A150	7.66 ± 0.29 ^a^	7.84 ± 0.22 ^a^	8.19 ± 0.32 ^a^	0.08 ± 0.03
	A150	9.86 ± 0.82 ^b^	9.03 ± 0.29 ^b^	7.66 ± 0.28 ^a^	−0.32 ± 0.05
	C-A300	7.75 ± 0.46 ^a^	6.58 ± 0.45 ^c^	8.22 ± 0.33 ^a^	0.10 ± 0.09
	A300	8.35 ± 0.26 ^a^	4.12 ± 0.35 ^d^	5.01 ± 0.10 ^e^	−0.50 ± 0.16
LEU–L-Leucine	C-A150	5.61 ± 0.28 ^a,d^	5.73 ± 0.33 ^a,d^	5.46 ± 0.22 ^a,d^	−0.02 ± 0.03
	A150	7.10 ± 0.39 ^b^	6.62 ± 0.37 ^b^	5.31 ± 0.23 ^a,d^	−0.26 ± 0.04
	C-A300	6.78 ± 0.87 ^b^	5.28 ± 0.19 ^a^	6.09 ± 0.32 ^d^	−0.09 ± 0.08
	A300	6.99 ± 0.16 ^b^	3.55 ± 0.21 ^c^	4.05 ± 0.16 ^c^	−0.41 ± 0.13
PHE–Phenylalanine	C-A150	4.89 ± 0.26 ^a,b^	5.07 ± 0.11 ^a,b^	3.42 ± 0.16 ^d^	−0.22 ± 0.05
	A150	4.73 ± 0.52 ^a,c^	4.06 ± 0.19 ^c,d^	5.31 ± 0.23 ^a,b^	−0.15 ± 0.04
	C-A300	5.48 ± 0.23 ^b^	4.93 ± 0.39 ^a,b^	4.09 ± 0.07 ^c,d^	−0.20 ± 0.04
	A300	5.52 ± 0.59 ^b^	3.48 ± 0.45 ^d^	3.49 ± 0.29 ^d^	−0.28 ± 0.09

TT—temporal trend. For each amino acid, average values sharing at least one common letter are not statistically different (*p* > 0.05).

**Table 5:** Content of five carotenoids in *Chlorella vulgaris* cultures in assays A150 (150 mM NaCl) and A300 (300 mM NaCl) and respective controls (with no salt added, C-A150 being the control for the assay at 150 mM NaCl and C-A300 the control for the assay at 300 mM NaCl) at days 8 (T8), 11 (T11), and 15 (T15) of the 15-day experiments.

Carotenoid	Assay	Carotenoid Content (mg g_dw_^−1^)			TT (mg g_dw_^−1^ d^−1^)
		T8	T11	T15	
Neoxanthin	C-A150	0.58 ± 0.02 ^a^	0.30 ± 0.05 ^b^	0.23 ± 0.02 ^f^	−0.044 ± 0.007
	A150	0.48 ± 0.05 ^b^	0.19 ± 0.02 ^e,f^	0.14 ± 0.02 ^e,g^	−0.048 ± 0.006
	C-A300	0.39 ± 0.03 ^c^	0.28 ± 0.03 ^d^	0.12 ± 0.01 ^g^	−0.039 ± 0.003
	A300	0.37 ± 0.05 ^c^	0.16 ± 0.02 ^e,g^	0.13 ± 0.02 ^e,g^	−0.026 ± 0.006
Violaxantin	C-A150	0.26 ± 0.02 ^a,d^	0.21 ± 0.02 ^a,c,e^	0.20 ± 0.03 ^c,e,g^	−0.009 ± 0.002
	A150	0.25 ± 0.03 ^a,d,e^	0.17 ± 0.02 ^c,g^	0.11 ± 0.02 ^f^	−0.021 ± 0.002
	C-A300	0.33 ± 0.06 ^b^	0.29 ± 0.03 ^d^	0.15 ± 0.01 ^f,g^	−0.027 ± 0.004
	A300	0.36 ± 0.05 ^b^	0.16 ± 0.01 ^c,f,g^	0.15 ± 0.01 ^f,g^	−0.022 ± 0.005
Lutein	C-A150	1.55 ± 0.22 ^a,d^	1.66 ± 0.08 ^a,d^	1.37 ± 0.13 ^d^	−0.029 ± 0.015
	A150	1.56 ± 0.16 ^a,d^	1.11 ± 0.21 ^c^	0.89 ± 0.10 ^c^	−0.094 ± 0.014
	C-A300	2.18 ± 0.24 ^b^	1.75 ± 0.21 ^a^	0.87 ± 0.05 ^c^	−0.193 ± 0.021
	A300	2.01 ± 0.30 ^b^	0.96 ± 0.11 ^c^	0.99 ± 0.18 ^c^	−0.106 ± 0.035
Zeaxanthin	C-A150	0.20 ± 0.02 ^a,d,e^	0.29 ± 0.02 ^b^	0.29 ± 0.04 ^b^	0.013 ± 0.003
	A150	0.29 ± 0.05 ^b^	0.16 ± 0.03 ^a,c,e^	0.24 ± 0.02 ^b,d^	−0.006 ± 0.005
	C-A300	0.21 ± 0.01 ^a,d^	0.22 ± 0.04 ^a,d^	0.14 ± 0.01 ^c,e^	−0.011 ± 0.003
	A300	0.12 ± 0.02 ^c^	0.12 ± 0.03 ^c^	0.17 ± 0.05 ^a,c,e^	0.010 ± 0.005
β-carotene	C-A150	0.12 ± 0.02 ^a^	0.09 ± 0.01 ^c^	0.05 ± 0.01 ^d^	−0.010 ± 0.001
	A150	0.13 ± 0.03 ^a^	0.07 ± 0.01 ^c,d^	0.05 ± 0.01 ^d^	−0.011 ± 0.002
	C-A300	0.21 ± 0.01 ^b^	0.14 ± 0.02 ^a^	0.07 ± 0.01 ^c,d^	−0.020 ± 0.002
	A300	0.21 ± 0.02 ^b^	0.12 ± 0.01 ^a^	0.07 ± 0.01 ^c,d^	−0.017 ± 0.002

TT—temporal trend. For each carotenoid, average values sharing at least one common letter are not statistically different (*p* > 0.05).

**Table 6:** Main results of the PCA application to the content of amino acids and carotenoids in the different assays.

Compound	PC1	PC2	PC3	PC4
ASP–Aspartic acid	**−0.939**	−0.058	−0.034	0.295
SER–L-Serine	**−0.897**	−0.410	0.131	0.061
GLU–Glutamic acid	**−0.961**	−0.076	0.034	0.183
GLY–L-Glycine	−0.484	**−0.823**	0.151	−0.034
HIS–L-Histidine	**−0.875**	0.117	0.042	0.451
THR–L-Threonine	**−0.887**	−0.333	0.210	0.183
ARG–Arginine	−*0.536*	0.203	**0.769**	0.006
ALA–Alanine	**−0.967**	−0.201	−0.035	0.126
PRO–L-Proline	−0.349	−0.077	**−0.883**	−0.172
TYR–L-Tyrosine	−0.261	**−0.909**	0.250	0.000
VAL–Valine	**−0.950**	−0.255	−0.070	−0.090
MET–Methionine	**−0.800**	−0.489	−0.128	−0.072
ILE–L-Isoleucine	**−0.718**	−0.358	0.343	0.370
LYS–Lysine	**−0.973**	−0.157	−0.005	0.128
LEU–L-Leucine	**−0.865**	−0.447	−0.142	−0.080
PHE–Phenylalanine	−0.287	**−0.936**	0.008	−0.080
Neoxanthin	−0.272	**−0.747**	−0.067	0.358
Violaxantin	−0.113	**−0.929**	−0.199	−0.006
Lutein	−0.135	**−0.936**	−0.102	0.174
Zeaxanthin	−0.326	−0.005	0.164	**0.891**
β-carotene	0.079	**−0.887**	−0.334	−0.229
**Eigenvalue**	12.31	4.64	1.87	1.03
**Variance (%)**	58.62	22.07	8.89	4.88
**Cumulative variance (%)**	58.62	80.69	89.59	94.47

Values in bold indicate the variables that mostly influence the correspondent principal component; factor loadings with absolute values greater than 0.5 are presented in italics.

## Data Availability

The dataset is available upon request from the authors.

## Supplementary Materials

The following supporting information can be downloaded at: https://www.mdpi.com/article/10.3390/bioengineering12030284/s1, Figure S1: Experimental setup for cultivation of *Chlorella vulgaris*; Figure S2: Observed pH of *Chlorella vulgaris* cultures in assays A150 (150 mM NaCl) and A300 (300 mM NaCl) and respective controls (no salt added) throughout an observed period of 15 days; Figure S3: Chromatogram of amino acid profile of *Chlorella vulgaris*. Peaks are labelled with their respective retention times and amino acid identities; Figure S4: Chromatogram of the carotenoid profile of *Chlorella vulgaris*. Peaks are annotated with their corresponding retention times and compound identities.
