# Supplementary material for: Amino Acid and Carotenoid Profiles of Chlorella vulgaris During Two-Stage Cultivation at Different Salinities
Source: Bioengineering (Basel). 2025 Mar 13;12(3):284. doi: 10.3390/bioengineering12030284 (PMC11939374; doi:10.3390/bioengineering12030284)
Supplement: Supplementary file 1 [file bioengineering-12-00284-s001.zip › bioengineering-3431875-supplementary.pdf]

Supplementary Material of

Amino Acid and Carotenoid Profiles of *Chlorella vulgaris* during Two-Stage Cultivation at Different Salinities

Ana S. Pinto[1,2], Carolina Maia[1,2], Sara A. Sousa[1,2], Tânia Tavares[1,2], José C.M. Pires[1,2,*]

[1] LEPABE – Laboratory for Process Engineering, Environment, Biotechnology and Energy, Faculty of Engineering, University of Porto, Rua Dr. Roberto Frias, 4200-465 Porto, Portugal.

[2] ALiCE – Associate Laboratory in Chemical Engineering, Faculty of Engineering, University of Porto, Rua Dr. Roberto Frias, 4200-465 Porto, Portugal.

*Corresponding author

Telephone: +351 22 041 3685

E-mail addresses: jcpires@fe.up.pt (J.C.M. Pires)

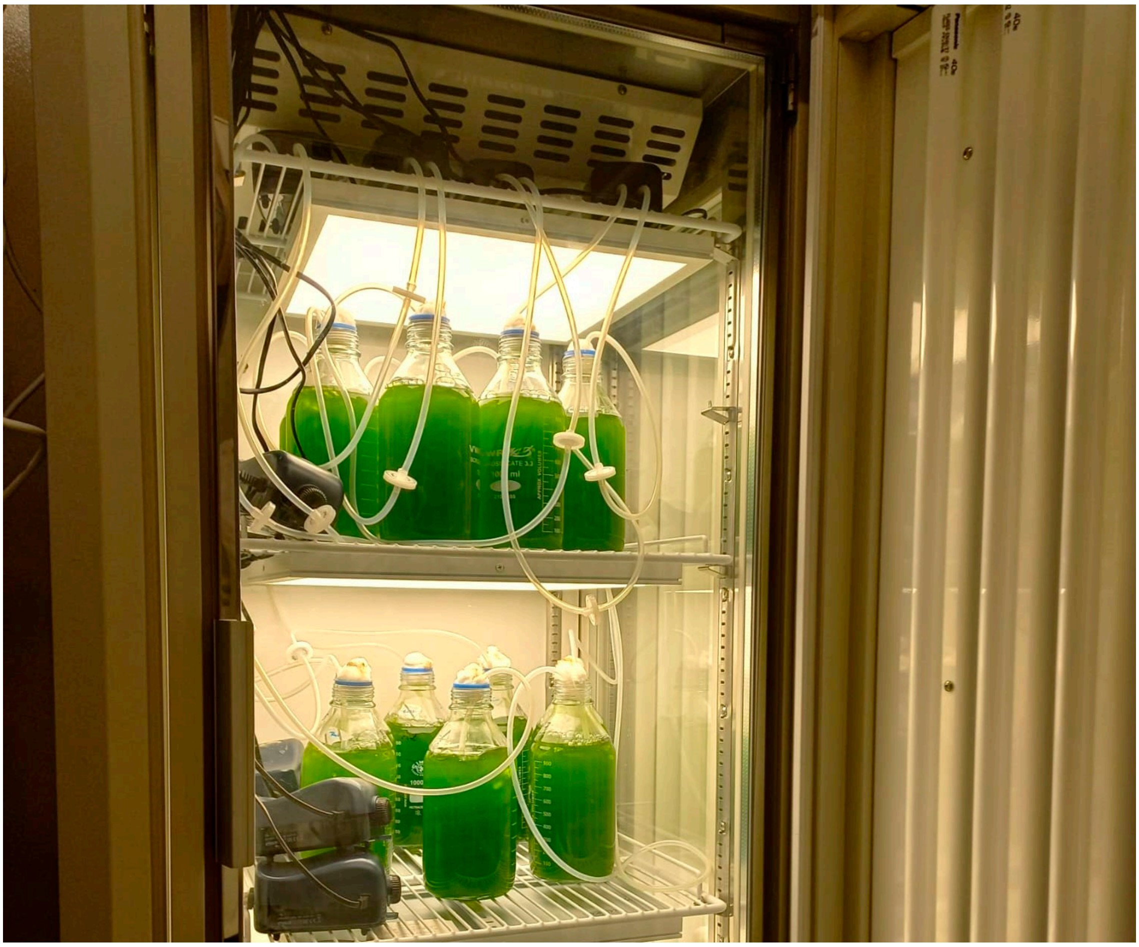

Figure S1. Experimental setup for cultivation of *Chlorella vulgaris*.

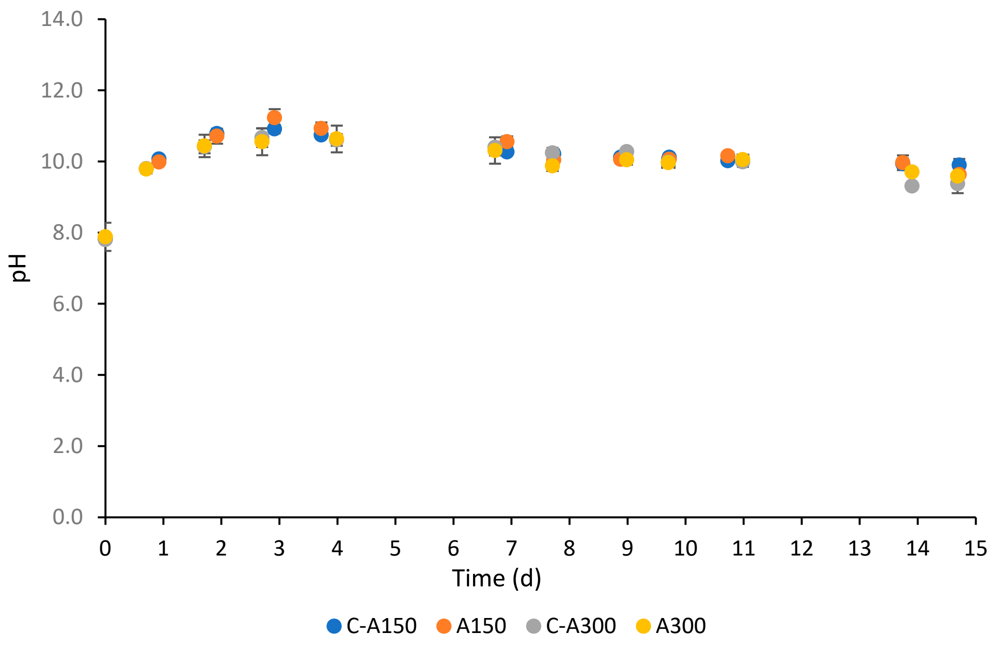

Figure S2. Observed pH of *Chlorella vulgaris* cultures in assays A150 (150 mM NaCl) and A300 (300 mM NaCl) and respective controls (no salt added) throughout an observed period of 15 days.

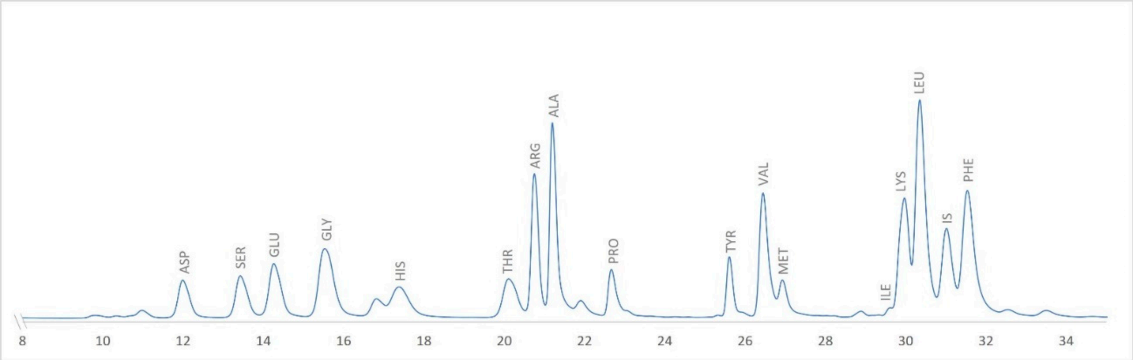

Figure S3. Chromatogram of amino acid profile of *Chlorella vulgaris*. Peaks are labelled with their respective retention times and amino acid identities.

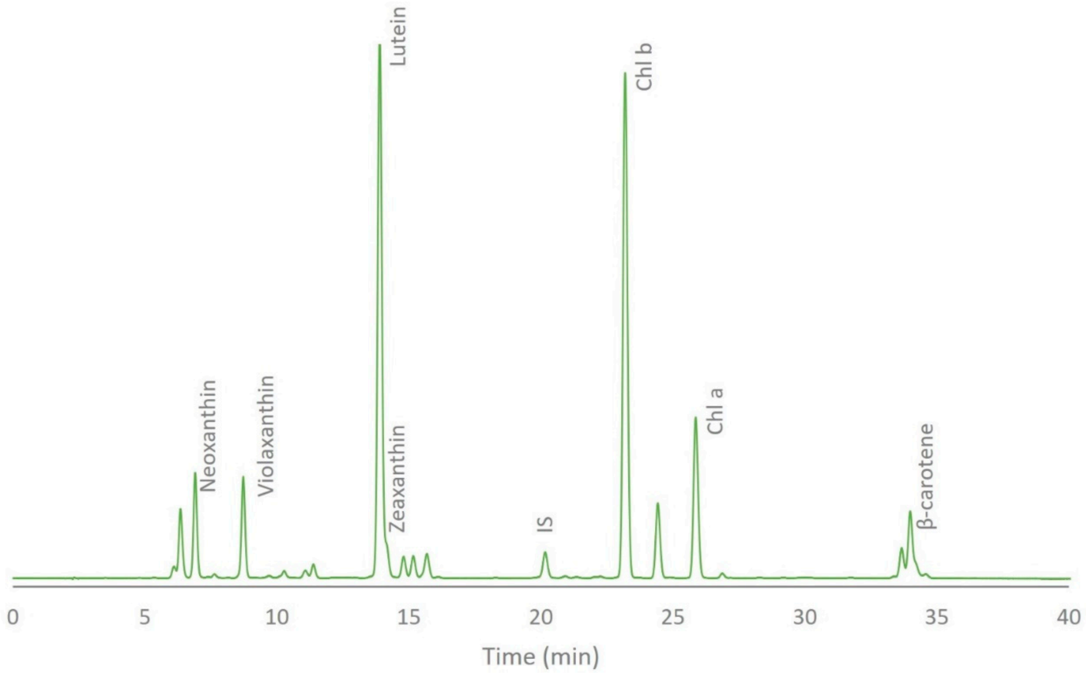

Figure S4. Chromatogram of the carotenoid profile of *Chlorella vulgaris*. Peaks are annotated with their corresponding retention times and compound identities.
